# New insights on the MMP-13 regulatory network in the pathogenesis of early osteoarthritis

**DOI:** 10.1186/s13075-017-1454-2

**Published:** 2017-11-10

**Authors:** Heng Li, Dan Wang, Yongjian Yuan, Jikang Min

**Affiliations:** 1 The First Affiliated Hospital of Huzhou Teachers College, Zhejiang Province, 313000 China; 2 Department of Orthopaedics, The First Affiliated Hospital of Huzhou Teachers College, The First People’s Hospital of Huzhou, Zhejiang Province, 313000 China

**Keywords:** Matrix metalloproteinases, MMP-13, Osteoarthritis, Non-coding RNA, DNA methylation, Autophagy

## Abstract

Osteoarthritis (OA) is the most common joint disorder and affects approximately half of the aged population. Current treatments for OA are largely palliative until the articular cartilage has been deeply damaged and irreversible morphological changes appear. Thus, effective methods are needed for diagnosing and monitoring the progression of OA during its early stages when therapeutic drugs or biological agents are most likely to be effective. Various proteinases involved in articular cartilage degeneration in pre-OA conditions, which may represent the earliest reversible measurable changes, are considered diagnostic and therapeutic targets for early OA. Of these proteinases, matrix metalloproteinase 13 (MMP-13) has received the most attention, because it is a central node in the cartilage degradation network. In this review, we highlight the main MMP-13-related changes in OA chondrocytes, including alterations in the activity and expression level of MMP-13 by upstream regulatory factors, DNA methylation, various non-coding RNAs (ncRNAs), and autophagy. Because MMP-13 and its regulatory networks are suitable targets for the development of effective early treatment strategies for OA, we discuss the specific targets of MMP-13, including upstream regulatory proteins, DNA methylation, non-coding RNAs, and autophagy-related proteins of MMP-13, and their therapeutic potential to inhibit the development of OA. Moreover, the various entities mentioned in this review might be useful as early biomarkers and for personalized approaches to disease prevention and treatment by improving the phenotyping of early OA patients.

## Background

Osteoarthritis (OA) is the most common joint disorder, affecting approximately half of the aged population (>65 years) and is characterized by the progressive degeneration of articular cartilage. The major clinical manifestation includes symptoms of knee pain, knee swelling, ankylosis, and limited activity [1]. OA results in mobility problems and severe pain during the intermediate or advanced stages and represents a leading socioeconomic burden in the developed world [2].

Currently, clinical diagnosis and monitoring of OA mainly rely on symptomatic and radiographic assessments and certain traditional laboratory tests [3–5]. Although their sensitivity and accuracy are relatively high, these methods fail to distinctively identify the developmental stages of OA. Similarly, the current treatments for OA are largely palliative until the articular cartilage has been deeply damaged and irreversible morphological changes have occurred; during the progression of OA the joints become completely dysfunctional and prosthetic replacement becomes necessary [6]. Thus, effective methods for diagnosing OA during its early stages are imperative and OA-related changes can likely be reversed by effective therapeutic drugs.

However, the development of disease-modifying drugs and the verification of their effectiveness in clinical trials are difficult to achieve due to the lack of a biomarker for the identification of patients with early OA-related changes. Articular cartilage damage is one of the most significant hallmarks of the early stages of OA [7]. Recently, studies have focused on the identification of biomarkers involved in articular cartilage degeneration in very early OA, which may represent the earliest reversible measurable changes.

Biomarkers that are related to the onset of articular cartilage degeneration during the early phase of OA include a number of matrix-degrading enzymes, such as the matrix metalloproteinase (MMP) family, the a disintegrin and metalloproteinase with thrombospondin type-1 motifs (ADAMTS) family, aggrecanases, etc. [8]. Of these proteinases, attention has focused on MMP-13 because it is significantly over-expressed in the joints and articular cartilage in patients with OA and can hardly be detected in normal adult tissues. MMP-13 is known to function as an extracellular matrix (ECM)-degrading enzyme in OA joints [9, 10]. In an experimental mouse OA model using a microsurgical technique, MMP-13 levels correlate with the presence of pathological chondrocytes that undergo hypertrophic differentiation in the early stage of OA development [11] and its over-expression can induce the onset of OA through excessive ECM degradation [12]. In contrast, OA progression is inhibited in MMP-13 knockout mice through protecting cartilage from proteoglycan loss and structural damage in an experimental OA model derived using medial meniscal destabilization surgery [13]. In clinical samples, MMP-13 was abnormally expressed during different stages of the OA process and was found to up-regulated during the early stage and down-regulated during the late stage in human OA cartilage [14]. Therefore, because MMP-13 is a central node in the cartilage degradation network [15], an understanding of the contribution of MMP-13 to the initiation/onset of OA is necessary. The activity of MMP-13 can be regulated at multiple levels [16], including epigenetic modification [17–19], transcriptional regulation, post-transcriptional regulation by ncRNAs [20, 21], and the activation or inhibition of proenzymes [9, 22]. In this review, we focus on new insights on the role of the MMP-13 regulatory network in the pathogenesis of early OA, considering transcriptional regulation [20, 21], different epigenetic alterations (such as DNA methylation and deregulation of non-coding RNA [17–19]), and autophagy [23]. We also discuss whether MMP-13 and its regulatory network could be useful in the diagnosis of early OA.

### Recent investigations into the role of MMP-13 in the onset of OA

The MMP family in humans comprises 24 different MMP genes and 23 different MMP proteins that are structurally related and characterized as zinc-dependent endopeptidases that degrade various components of the ECM and basement membrane [24]. Based on their domain organization, their sequence similarities, and the specificity of their substrates, the MMPs can be classified into the following four groups: gelatinases, matrilysins, archetypal, and furin-activated. The archetypal MMPs can be classified into the following three subgroups according to their substrate specificities: collagenases, stromelysins, and other archetypal MMPs [25]. MMP-13, also known as collagenases-3, is a member of the collagenase subgroup [25].

Recently, the mRNA and protein expression of MMP-13 were shown to be increased during the process of OA onset. In rat initial/onset OA models, the increased expression of MMP-13 was detected using both immunohistochemistry and qRT-PCR, suggesting that MMP-13 was a factor responsible for early-onset OA [26, 27]. A similar result was observed in anterior cruciate ligament transection (ACLT) or ACLT with partial medial meniscectomy (ACLT + MMx) rat OA models in which a significant increase in the mRNA levels of MMP-13 was observed as early as the first week post-surgery, and the increased expression remained elevated throughout the 10-week study [28].

In addition to MMP-13, its regulatory factors, including interacting protein of MMP-13, were involved in the process of OA onset. For example, low-density lipoprotein receptor-related protein 1 (LRP1) binds both pro- and activated MMP-13, and is a key modulator of the extracellular levels of MMP-13. LRP1 was associated with the function of MMP-13 in the physiological turnover of the ECM [17]. Thus, MMP-13 plays a key role in the initiation of the shift from normal chondrocytes to the pathological phase, at least partially, by driving the shedding of LRP1 in cartilage, which may help us develop a new approach for controlling the onset of OA. A similar function was observed in leptin-targeted gene therapy. Leptin, which is a 16-kDa non-glycosylated protein product of the obese (ob) gene, exhibits a detrimental effect on articular cartilage. Small interference RNA against leptin could directly deactivate MMP-13 in the OA chondrocyte and possibly has therapeutic potential for OA treatment [29]. Similarly, high temperature requirement A1 (HTRA1) is increased in both human OA cartilage and the articular cartilage in mouse models of OA [30]. Once HTRA1 activity disrupts the pericellular matrix, which may occur early before the overt symptoms of OA develop, chondrocyte receptors such as DDR2 may be activated by type II collagen in the fibrillar form, leading to the preferential up-regulation of MMP-13 and further degradation of the interterritorial matrix [31].

Another kind of protein that interacts with MMP-13 functions by activating the latent form of the MMP-13 protein. Activators of pro-MMP-13 are potential therapeutic targets for early OA since activation of the zymogen form of MMP-13 occurs relatively early in the OA course. Recently, Magarinos et al. [32] constructed an ex vivo mouse femoral head explant system and studied the effects of tryptase-β and MCP-6 on MMP-13 enzymatic activity. The results show that tetramer-forming tryptases initiated aggrecanolysis by proteolytically activating the latent proenzyme form of MMP-13. There is specificity as to which neutral proteinase zymogen of MMP-13 in the joint is susceptible to tryptase-dependent activation, although no evidence of direct interaction between tryptase-β and/or MCP-6 with latent pro-MMP-13 has so far been provided [32].

Furthermore, the function of MMP-13 in the onset/initiation of OA may be mediated by specific signaling pathways. Many transcription factors are involved during different stages of OA, such as LEF1, NF-κB, ELF3, HIF2α, and Runx2, and most transcription factors directly or indirectly impact MMP-13 transcription. For example, Yun et al*.* [33] found that LEF1, interacting with β-catenin, directly binds the 3′ region of the MMP13 gene and transactivates MMP13 promoter activity, possibly through a change of DNA conformation. SIRT1 represses MMP-13 in human OA chondrocytes, which appears to be mediated, at least in part, through repression of the transcription activity of LEF1. In the SIRT1 knockout (KO) mouse model, LEF1 and MMP-13 appeared elevated in the superficial zone of articular cartilage, which suggested the initiation of OA [34]. NF-κB activation results in the activation of ELF3 and HIF2α, which leads to activation of MMP-13 and facilitates the shift of normal articular chondrocytes to a hypertrophic-like differentiated state, subsequently initiating OA onset [35].

In addition, MMP inhibitors could be developed to control the onset of OA. For example, Wang et al. [36] and Julovi et al. [37] investigated the effects of high molecular weight hyaluronic acid (HMW-HA) on the gene expression of 16 OA-associated cytokines and enzymes. These authors found that HMW-HA has a structure-modifying effect on early OA by effectively inhibiting the production of MMP-1, MMP-3, and MMP-13 in human articular cartilage.

Mechanical stress also plays a key role in the pathogenesis of OA cartilage destruction and MMP-13 has been proven to be involved in the early stage in a series of in vivo and in vitro experiments. For example, Kamekura et al. [11] created a mechanical stress-induced OA mouse model and found MMP-13 was markedly induced and colocalized in the early stage OA cartilage in vivo. Subsequently, studies have demonstrated that mechanical stress upregulated MMP-13 expression rapidly in chondrocytes in vitro: cyclic tensile stress (CTS) induced MMP-13 expression in rat cultured normal chondrocytes. The upregulation of MMP-13 was observed within 3 h, which was earlier than that of IL-1β [38]; a static load exceeding 40 psi initiated extracellular matrix degradation through an increase of catabolic MMP-13 encoding gene expression within 24 h [39]. In addition, MMP-13 genes were also significantly enhanced when chondrocytes were co-cultured with excessively mechanically stressed osteoblasts [40]. These results demonstrate that alterations in cartilage metabolism can be induced by stressed chondrocytes and osteoblasts through a MMP-13-dependent pathway, indicating a possible explanation for the onset and progression of OA.

**Fig. 1:**
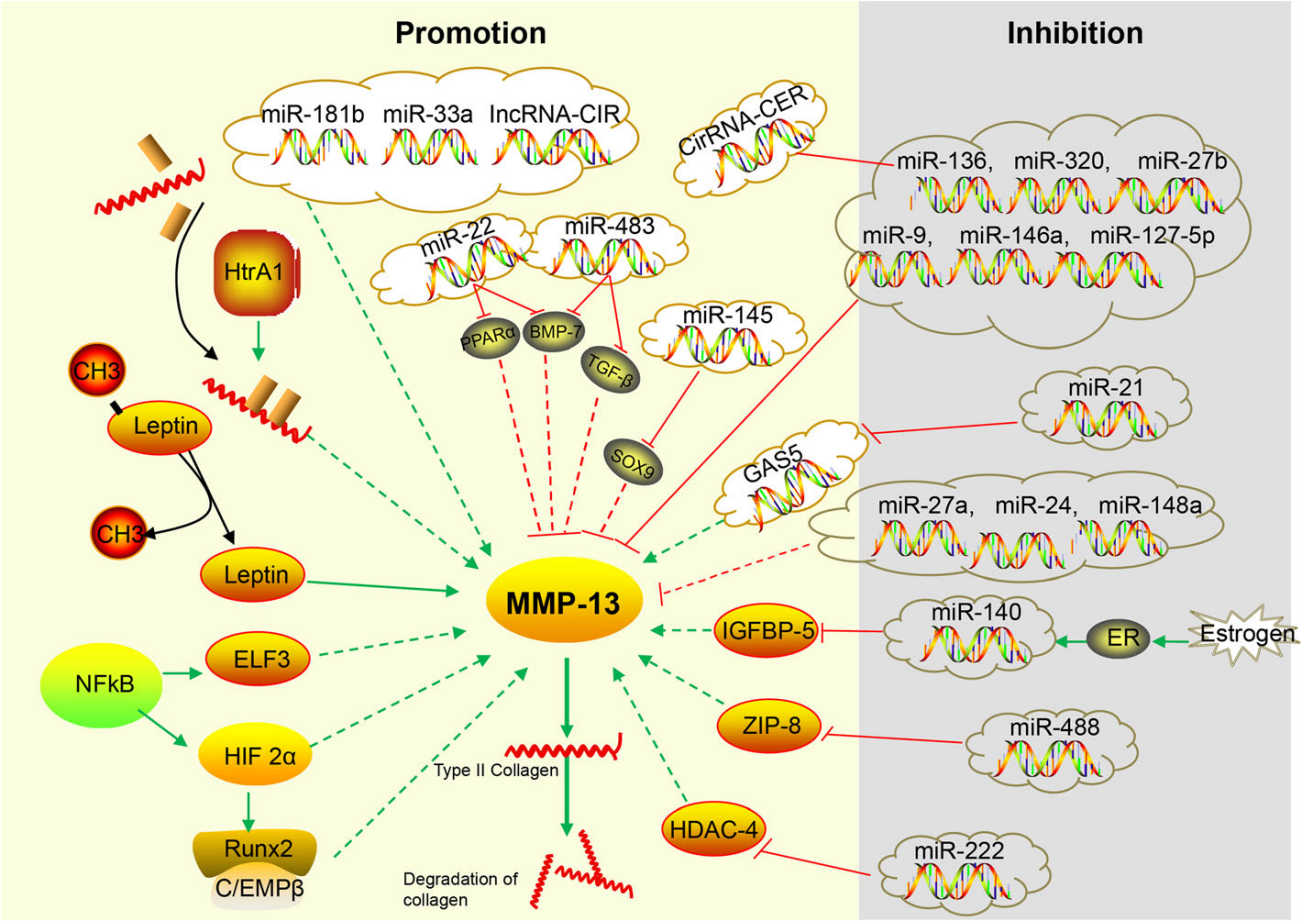
Regulatory network of MMP-13 in OA chondrocytes. In the illustration, the *dotted lines* indicate an indirect effect on MMP-13 and the *solid lines* indicate a direct effect on a downstream target. *Green lines* indicate promotion and *red lines* inhibition of MMP-13. The *yellow background* indicates increasing expression level and the *gray background* decreasing expression level in OA chondrocytes

Although CTS is known to upregulate MMP-13 expression via the Runx-2/Cbfa1 [41] and NF-κB [42] pathways, the detailed regulatory mechanisms of mechanical stress on MMP-13 remain unknown. It has long been known that the integrin signal pathway serves as a mechanotransducer in chondrocytes by “integrating” the extracellular matrix with cytoskeletal structures and signals in response to mechanical forces. Its effect on MMP-13 could partially account for the regulatory mechanisms of mechanical stress on MMP-13 [43]. For instance, the matrix protein fibronectin fragment (FN-f), which is generated by the action of MMP-13, stimulates chondrocytes to produce MMP-13 through binding with α3β1, α4β1, α5β1, and αVβ1 integrins [43]. Similarly, angiopoietin-like 4 (ANGPTL4) promotes ECM degradation through induction of MMP-13 by binding integrins β1 and β5 and modulating integrin-mediated signaling [44]. Thus, MMP-13 is a well-known key player in the pathology of early OA due to its capacity to directly or indirectly initiate the degradation of a wide range of downstream matrix and collagen components via its regulatory factors through specific signaling pathways (Fig. 1). A few of its regulatory factors and inhibitors could be explored as potential targets for therapeutic interventions in early OA.

### Role of ncRNA-mediated MMP-13 regulation in early OA

The human transcriptome includes many transcripts without protein-coding potential, namely ncRNAs. The deregulation of ncRNAs is closely associated with diverse diseases, including OA [45]. MicroRNAs (miRNAs) are single-stranded ncRNA molecules that are typically 19 to 25 nucleotides long. Recently, alterations in the expression levels of miRNAs have been linked to a variety of disease processes and provide a new horizon in OA [12]. In fact, several differentially expressed miRNA profiles were identified in normal articular cartilage and mild to severe OA auricular cartilage [46]. Certain miRNAs were considered suppressors or promoters of the early steps in the chondrogenic program [47]. Due to the significant role of MMP-13 in promoting the initiation of OA, an understanding of the miRNA-mediated regulatory network of MMP-13 is necessary for the identification of valid alternative therapeutic approaches for the early disease process.

Jones et al*.* [48] compared the miRNA expression profiles in human cartilage and bone between late-stage OA patients and normal donors. In this study, 17 miRNAs in the cartilage and 30 miRNAs in the bone showed differential expression greater than fourfold in the diseased tissue compared to that in the normal tissue [48]. Among the miRNAs, miR-9 was subsequently shown to directly target MMP-13. miR-9 was up-regulated in late-stage OA cartilage and bone samples compared with normal specimens. The over-expression of miR-9 in isolated chondrocytes decreased the secretion of MMP-13, while the inhibition of miR-9 increased the levels of this metalloproteinase. Consistently, MMP-13 has been shown to be down-regulated in late-stage human OA cartilage, which was based on an experiment in which MMP-13 showed a higher gene expression level in the intact region of the OA cartilage than in the damaged region of the OA cartilage [14]. Furthermore, miR-9 was found to inhibit the secretion of collagen type II in isolated human chondrocytes, which was based on the mechanism of targeting MMP-13 [48]. Due to a shortage of early OA samples, however, the variations in the expression of miR-9 and MMP-13 during OA onset could not be explored.

In addition to miR-9, four other miRNAs, miR-146a, miR-127-5p, miR-27b, and miR-320, target MMP-13 in chondrocytes. Okuhara et al. [49] investigated the relative expression levels of miR-146a in peripheral blood mononuclear cells derived from OA patients with different K-L grades (Kellgren and Lawrence grading scale). The expression level of miR-146a at grade 0 was significantly lower than those at K-L grades 2, 3, and 4. However, the expression level of miR-146a decreased as the K-L classification grade increased. Therefore, the expression of miR-146a was significantly higher during the early stages of OA than during the later stages [49]. Similar results were observed in cartilage samples derived from patients with different grades according to a modified Mankin scale [50]. Yamasaki et al*.* [50] found that miR-146a was significantly higher in cartilage in patients with grade I OA and lower in cartilage in patients with grade II and III OA. Furthermore, the variation in the expression of miR-146a in cartilage in patients with grade II OA was inversely related to the expression level of MMP-13. Specifically, the expression of MMP-13 was significantly increased in grade II OA [50] but subsequently decreased in the grade III OA cartilage, which is consistent with the level of miR-146a. Further studies have demonstrated that miR-146a suppressed the expression of aggrecan, Col2, MMP-13, and ADAMTS-5 in human chondrocytes [51] and functions as a suppressor of autoimmunity and myeloproliferation and particularly as a negative feedback regulator of MMP-13 [52]. Notably, during certain stages of OA, such as in grade I and III patients, the expression of MMP-13 was not entirely reversed by miR-146a. This discrepancy can be explained by two aspects: First, the clinical samples differ from OA cell models. The expression levels of MMP-13 and miR-146a in various clinical samples were affected not only in the chondrocytes but also in synovial cells and their microenvironments, which cannot be anticipated as a perfect one-to-one correspondence. Second, Yamasaki et al. assayed the mRNA expression of MMP-13 using only RT-PCR [50]. However, miRNAs can regulate their targets at the post-transcription level, such as by inhibiting the translation process. Therefore, further assays should be performed to detect MMP-13 protein levels.

Park et al*.* [20] found a significant reduction in miR-127-5p expression in OA cartilage compared with normal cartilage. The up-regulation of MMP-13 expression by IL-1β was correlated with the down-regulation of miR-127-5p expression in human chondrocytes. MMP-13 has been shown to function as a direct target of miR-127-5p [20] and miR-27b is also a direct negative regulator of MMP-13, as shown by several research groups. Akhtar et al*.* [53] investigated the expression of 352 human miRs in chondrocytes stimulated with IL-1 and identified 44 significantly differentially expressed miRs. Among these miRs, miR-27b was down-regulated by threefold in the IL-1β-stimulated OA chondrocytes compared with in the unstimulated OA chondrocytes. In the OA cell model, the expression of MMP-13 was inversely correlated with miRNA-27b expression [53]. miR-27b targets MMP-13 mRNA and is suppressed by mitogen-activated protein kinase (MAPK) and NF-kB signaling [53]. Recently, Meng et al*.* [54] reported that over-expression of miR-320 suppressed the activity of a reporter construct containing the 3′ UTR and inhibited MMP-13 expression in IL-1β-treated primary mouse chondrocytes.

Several other miRNAs that are down-regulated in OA also indirectly inhibit MMP-13 expression, including miR-27a, miR-140, miR-488, miR-24, miR-148a, and miR-222, and these miRNAs were decreased in OA chondrocytes. miR-27a may indirectly regulate the levels of MMP-13 and proanabolic insulin-like growth factor binding protein (IGFBP)-5 by targeting the upstream effectors of both genes [55]. Similarly to miR-27a, miR-140 is reduced in OA tissue and its functions include the indirect regulation (through post-transcriptional inhibition) of IGFBP-5 and MMP-13 [55]. 17-β-Estradiol (E2) has been recently shown to suppress the expression of MMP-13 in human articular chondrocytes, which was accompanied by an up-regulation of the expression of miR-140. Furthermore, the estrogen receptor (ER) directly binds the miR-140 promoter, and estrogen acts via the ER and miR-140 pathway to inhibit the expression of MMP-13. Therefore, the ER/miR-140/IGFBP/MMP-13 signaling pathway may be a potential target for therapeutic interventions for OA patients. In addition, more potential targets have been reported. For example, a decrease in the expression of miR-488 was observed in OA chondrocytes and miR-488 inhibits MMP-13 activity by targeting ZIP-8 [56]. Philipot et al*.* [15] reported that the down-regulation of miR-24 was consistent with the increased production of MMP-13 in human OA chondrocytes. Chondrocytes from OA patients also showed a decrease in the expression of miRNA-148a, while its over-expression inhibited the presence of MMP-13. Consequently, different approaches that increase miRNA-148a have been suggested to inhibit chondrocyte hypertrophy [57]. miR-222 was significantly down-regulated in OA chondrocytes [58] and its over-expression was accompanied by the down-regulation of HDAC-4 and MMP-13 levels. HDAC-4 has been shown to be a direct target of miR-222; the treatment of chondrocytes with the HDAC inhibitor trichostatin A (TSA) suppressed the MMP-13 protein level, whereas the over-expression of HDAC-4 displayed the opposite effects. The introduction of miR-222 into the cartilage of medial meniscus-destabilized mice significantly reduced the cartilage destruction and MMP-13 level. Altogether, miR-222 may be involved in cartilage destruction by targeting HDAC-4 and regulating the MMP-13 level [58].

However, several miRNAs are up-regulated in OA and indirectly increase MMP-13 expression, including miR-22, miR-33a*,* miR-181b, miR-145, and miR-483. Iliopoulos et al*.* [59] measured the expression of 365 miRNAs in articular cartilage obtained from OA patients and normal individuals. These authors identified nine up-regulated miRNAs and seven down-regulated miRNAs in the OA cartilage. Of these miRNAs, the inhibition of endogenous miR-22 blocks MMP-13 activity by up-regulating bone morphogenetic protein (BMP)-7 and peroxisome proliferator activated receptor alpha (PPARα) expression [59]. miR-181b was significantly up-regulated in OA chondrocytes and the use of an inhibitor to attenuate miR-181b can reduce MMP-13 expression [60]. Similarly to miR-181b, the expression of miR-33a is increased in OA chondrocytes and exogenous miR-33a significantly elevated MMP-13 expression levels [61]. miR-145 is also increased in OA chondrocytes and its over-expression increased MMP-13 expression. The effect of miR-145 on MMP-13 may be mediated by Sox9, i.e., miR-145 negatively regulates endogenous Sox9 by directly targeting Sox9 in human articular chondrocytes, while MMP-13 was down-regulated in Sox9-over-expressing hypertrophic chondrocytes [62]. The expression of miR-483 was significantly up-regulated in an operative murine model of OA, particularly one week after surgery, suggesting that miR-483 may play critical roles in the early pathogenesis of OA. The expression of miR-483 was negatively correlated with the mRNA expression of BMP-7 and TGF-β and positively correlated with MMP-13 according to a Pearson correlation analysis [63].

Although studies investigating miRNAs have dominated the field of RNA biology in recent years, multiple studies have indicated that long non-coding RNAs (lncRNAs) are involved in a variety of biologic processes. The lncRNAs have been defined as ncRNAs of < 200 nucleotides in length and are characterized by the complexity and diversity of their sequences and mechanisms of action [64, 65]. The deregulation of lncRNA is closely associated with the OA process [45]. Regarding the differential expression profiles of lncRNAs in the process of OA, up to 152 lncRNAs have been found to be differentially expressed (more than eightfold) between OA and normal cartilage. lncRNA-CIR was particularly over-expressed in OA cartilage compared with in normal cartilage. The expression of lncRNA-CIR is increased in OA tissues, which is consistent with the up-regulation of MMP-13. Furthermore, the silencing of lncRNA-CIR reduced the expression of MMP-13 and vice versa, suggesting a co-regulation of lncRNA-CIR and MMP-13 in articular cartilage [66]. GAS5 is another lncRNA that was up-regulated in OA chondrocytes compared with non-OA and normal chondrocytes [67]. Furthermore, GAS5 was identified as a direct target of miR-21 and the over-expression of GAS5 was subsequently found to promote OA pathogenesis by increasing MMP-13 expression levels [67].

In addition, circular RNAs (circRNAs) are a newly reported family of ncRNAs that function as miRNA “sponges” that naturally sequester and competitively suppress miRNA activity. Liu et al*.* identified 71 circRNAs that were differentially expressed between OA and normal cartilage using an Arraystar Human circRNA Array. CircRNA-CER was confirmed to be over-expressed in OA, which is consistent with the up-regulation of MMP-13. It was also shown to act as a decoy for MMP13 by functioning as a competing sponge of miR-136. The sequence of the circRNA-CER 3′ UTR matches miR-136, and MMP-13 is the direct target of miR-136 [68].

In summary, considering the critical role of MMP-13 in the onset of cartilage degradation, clarifying the regulatory network of ncRNA-mediated MMP-13 is critical for an understanding of the pathogenesis of OA and exploring new potential diagnostic and therapeutic targets (Fig. 1).

### Role of DNA methylation in MMP-13 gene expression in OA

DNA methylation, which is one of the most well-clarified epigenetic changes, targets DNA sequences by adding a methyl (CH3) group to the carbon 5 (C5) position of cytosine and results in the phenotype of gene silencing [69–71]. These modified sites are high-density CpG regions, namely CpG islands, which are typically located in the gene promoter regions [72, 73]. More importantly, while DNA methylation is heritable at the cellular level, it is potentially reversible. Therefore, the DNA methylation status could be a new molecular target for OA progression, particularly during the early stages of the disease [74].

Several genome-wide DNA methylation studies involving OA patients found many differentially methylated CpG sites in promoters of genes associated with OA development [75–77], which suggested that DNA methylation modifications play an important role in the development of OA [29, 77, 78]. In these studies, promoter hypomethylation events were associated with the increased expression of several MMPs involved in cartilage degradation [74, 79]. For example, Roach et al*.* [79] first compared the DNA methylation status of four degradative enzymes (i.e., MMP-3, MMP-9, MMP-13, and ADAMTS-4) between OA and non-OA samples. Of the MMPs, MMP-13 was the most heavily methylated in the non-OA samples (95.8%), and its methylation rate decreased to 79.8% in OA samples. The authors identified that both the −134 and −110 sites in the MMP-13 promoter became demethylated during the OA process, even at the early stage. Subsequently, the −104 CpG site in the MMP-13 promoter was also shown to be consistently demethylated and correlated with increased MMP-13 expression. cAMP response binding element (CREB) was identified as a regulating factor that is able to bind the MMP-13 promoter only when the CpG −104 is demethylated [80]. Subsequently, the demethylation of specific CpG sites at −110 bp in the MMP-13 promoter was observed in chondrocytes derived from human OA cartilage, which strongly correlated with higher levels of MMP-13 expression. The methylation status resides within a HIF consensus motif, which results in the most marked suppression of MMP-13 activities. In chromatin immunoprecipitation assays, the methylation of the −110 CpG site in the MMP-13 promoter inhibited its HIF-2α-driven transactivation and decreased HIF-2α binding to the MMP-13 proximal promoter, which may attenuate the process of OA [81]. Recently, Moazedi-Fuerst and colleagues [82] performed a genome-wide methylation screening to identify potential differences between paired mild and severe OA human cartilage. However, the authors could not confirm the presence of differential methylation of MMP-13 in OA, which may be because their “target probe set” did not cover the MMP-13 promoter. Therefore, the CpG sites −104, −110, and −134 are demethylated in OA cartilage and are correlated with elevated MMP-13 expression and cartilage destruction. The highly novel link between the epigenetic status of MMP-13 and OA development may help develop a new strategy to treat early OA [71].

DNA methylation is involved in the MMP-13-driven OA process not only through directly targeting the MMP-13 promoter, but also by targeting the promoters of genes encoding MMP-13-mediated proteins. For example, RunX2 cooperates with the CCAAT/enhancer binding protein β to drive MMP-13 transactivation, because the protein complex of Runx2 and C/EBPβ is located between −144 and −89 bp of the MMP-13 promoter (which contains a C/EBPβ-binding motif at residues −103 to − 97 and a RunX2 binding motif at −138 to −132). HIF-2α is a transcriptional inducer of C/EBPβ in chondrocytes and is located between −103 and −46 bp of the C/EBPβ promoter [83]. Therefore, HIF-2α regulated MMP-13 expression not only by directly binding the MMP-13 promoter but also by binding the upstream protein promoter of MMP-13. Jeffries et al*.* [76] performed a genome-wide DNA methylation study to identify the DNA methylation changes in OA cartilage tissue and identified that RUNX2 was hypomethylated, which may result in high expression of its protein product and further promote the transcriptional activity of MMP-13 in OA. In addition, the Ingenuity Pathways Analysis (IPA) system (Ingenuity Systems) identified miR-27a to be enriched among the differentially methylated genes, which linked to MMP-13 in vitro [55]. DNA methylation was also involved in the regulation of leptin expression in OA, which affects its downstream target, MMP-13 [29].

**Fig. 2:**
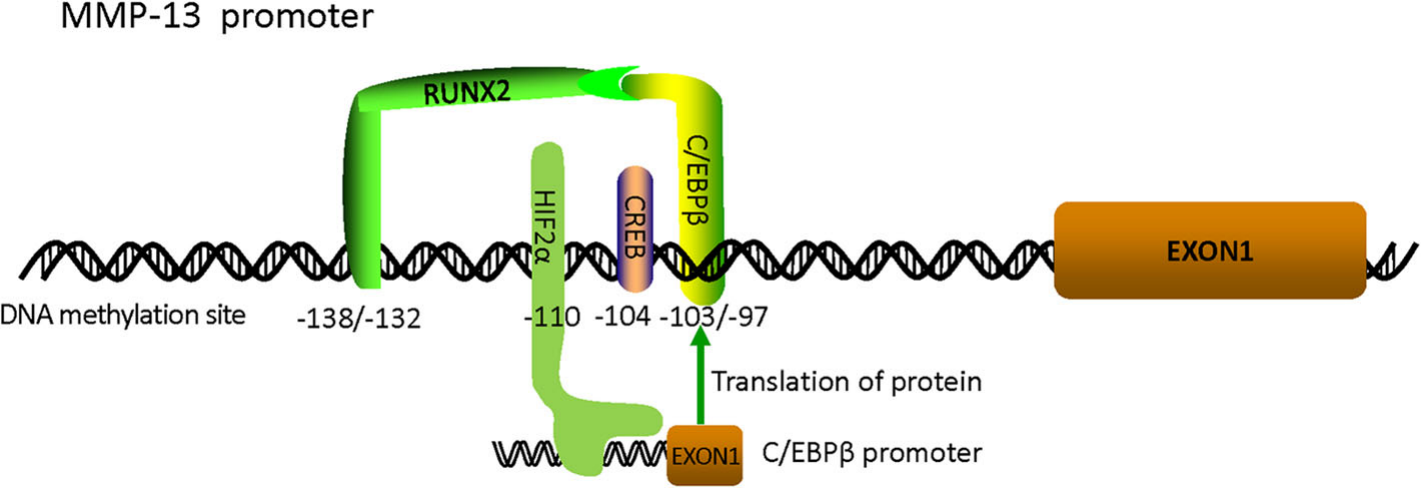
Model for DNA methylation of the MMP-13 promoter. CREB binds to the MMP-13 promoter when the −104 CpG is demethylated. HIF-2α binds to the MMP-13 promoter when the −110 CpG is demethylated. C/EBPβ and RunX2 bind between base pairs −103 and −97 and −138 and −132, respectively, and cooperate to promote MMP-13 mRNA transcription. At the same time, HIF-2α promotes C/EBPβ mRNA transcription by binding between base pairs −103 and −46 of the C/EBPβ promoter

Altogether, MMP-13 expression was directly or indirectly regulated by epigenetic mechanisms during early OA (Fig. 2), suggesting that DNA methylation could be a new target for the treatment and diagnosis of early OA.

### Relationship between MMP-13 and autophagy

The wide range of MMP-13 proteolytic capacities suggests that it is a powerful, potentially destructive proteinase; thus, it was believed for a long time that MMP-13 is not produced in most adult human tissues in the steady state. However, recent studies have revealed that human chondrocytes isolated from healthy adults constitutively express and secrete MMP-13, but MMP-13 is rapidly endocytosed and degraded by chondrocytes, which is suggestive of the key role of autophagy in the regulation of the MMP-13 protein [17]. Autophagy is a cellular self-protection mechanism that removes damaged organelles and intracellular unfolded proteins [84]. During this process, the expression levels of several autophagy regulators, including autophagy-related genes (Atgs), Beclin-1, and the LC3-II/LC3-I ratio, are increased. Recently, a few studies have suggested that autophagy protects against cartilage degradation under MMP-13-mediated OA conditions [5–87].

Firstly, in some OA models, researchers found that the patterns of variation of autophagy levels and MMP-13 expression levels were reversed. For instance, during the insulin-induced OA pathological process, the increased MMP-13 expression was consistent with the reduced LC3 II expression as well as the decreased autophagy [88, 89]. In a computational model of aging OA established by Hui et al., an increase in the MMP-13 levels was accompanied by a gradual decline in lysosome activity and Bcl-2 levels, which was similar to the experimental data [90]. Furthermore, the process of age-associated spontaneous OA can be accelerated by the loss of the von Hippel-Lindau (VHL) gene in adult articular cartilage, which was illustrated by an earlier study on MMP-13 over-expression and compromised chondrocyte autophagy [91]. The over-expression of GAS5 in articular chondrocytes was another novel method of establishing an OA model, which consistently showed increasing MMP-13 levels and suppressed autophagy responses during OA pathogenesis [67].

Secondly, MMP-13 expression levels were changed when autophagy activities were inhibited or stimulated in chondrocytes. For example, sucrose treatment and Torin 1 (a chemical autophagy inducer) treatment can induce autophagy and significantly inhibited the mRNA expression of MMP-13 in human OA chondrocytes [92–94]. In contrast, 3-methyladenine (a chemical autophagy inhibitor) treatment induced the loss of autophagy, which is linked to increased MMP-13 mRNA expression and the development of OA [93, 94]. In addition, Bouderlique et al. [95] generated mice which lack the Atg5 gene in their chondrocytes (Atg5cKO). Development of OA was observed in Atg5cKO mice, associated with an increase in MMP-13 levels in the articular cartilage.

In conclusion, current studies report opposite variation tendencies between autophagy and MMP-13 levels in OA models as well as in autophagy-regulated models, which implies that autophagy may play a protective role in the pathogenesis of OA by inhibiting MMP-13 production. However, closer links between autophagy and MMP-13 levels in OA progression need far more supporting evidence. For example, whether or how will autophagy change when MMP-13 activities are inhibited or stimulated in chondrocytes? Thus, the detailed molecular mechanism and effect of MMP-13 on autophagy should be further explored.

## Discussion

Because abnormal MMP expression levels have been linked to OA progression, the MMPs are attractive targets for the development of specific inhibitors that may have clinical applications. In a model of explanted human OA cartilage, an MMP inhibitor targeting MMP-13 could block the ECM degradation in OA cartilage [96]. Therefore, certain synthetic MMP-13 inhibitors have been developed as promising agents to treat OA [97]. To the best of our knowledge, however, few MMP-13 inhibitors have been successfully utilized as therapeutic agents thus far. Although many factors may have contributed to this failure of MMP inhibitors in the clinic, we identified two possible reasons in this review.

First, as we mentioned in another review [25], MMP-13 shares generally similar active site structures with other members of the MMP family, has overlapping specificities, and plays numerous key roles in important biological processes other than OA development; therefore, designing MMP inhibitors that are highly selective and have low side effect profiles is challenging.

Therefore, to improve the selectivity of therapeutic agents for OA therapy, new therapeutic agents targeting MMP-13 should be able to inhibit MMP-13 expression indirectly by targeting key central nodes in its interaction network rather than targeting the MMP-13 protein. Therefore, DNA methylation sites and ncRNA-mediated MMP-13 expression are potential promising targets for selectively inhibiting MMP-13 expression, without interfering with the structural similarities of the MMP catalytic domains. Moreover, epigenetic regulators can target multiple molecules, frequently in the context of a network, which makes them extremely efficient at regulating distinct biological processes that are relevant to the OA process [25].

Second, all clinical trials conducted to date involved patients with stage III–IV OA, and several overlapping pathways may contribute to the irreversible and uncontrolled cartilage degradation. Therefore, targeting MMP-13 and its regulators for early detection and intervention in OA could be feasible because the process of cartilage degradation is irreversible and the onset mechanism of OA is relatively controllable.

It should be noted that current knowledge on MMP13 expression regulation is still limited and the specific roles of MMP-13 during different stages of joint degeneration also should be further explored, although they have been focused on for more than 20 years. For example, a close correlation between MMP-13 expression and osteophyte development was noted by several studies [28, 98, 99]. However, how to explain the observation that MMP-13-deficient mice are not resistant to osteophyte development [13]? We try to explain it based on the speculation that MMP-13 may play a key role in the early OA process, mainly degrading ECM and cartilage, while osteophytes develop in the late stage when MMP-13 has less function in joints. During the late stage, although MMP-13 is still expressed in the majority of osteophyte tissues, its function can be substituted for by other factors in its regulatory network. Anyway, the speculation should be proved or revised by more evidence; therefore, more detailed research on MMP-13 function should be done during different OA stages and in more types of joint tissues, including not only chondrocytes or articular cartilage but also synovial fibroblasts, synovial mast cells, subchondral bone, and osteophytes.

## Conclusions

In this review, we discuss how MMP-13-mediated regulation may improve or inhibit the onset of OA through the functions of interacting factors, the autophagy process, and epigenetic modification (Table 1). Multiple regulatory pathways are involved in MMP-13-mediated regulation, and many of these pathways remain unknown. Based on the growing relevance of the autophagy process and epigenetic modification in the regulation of the OA process, it is likely that further work in this field will reveal additional interacting factors that can modulate MMP-13, thus contributing to their functional regulation in the different physiological and pathological contexts. A thorough understanding of MMP-13-mediated regulatory mechanisms governing OA onset and development should provide new insights into the diagnosis and treatment of early OA.

**Table 1:** Functional and pathological implications of MMP regulation in OA

Regulatory factor	Direct/indirect target of MMP-13	Effect on MMP-13	Function in OA onset/progression
LRP1 [17]	Directly binds to MMP-13	Endocytosed and degraded MMP-13	Inhibits OA onset
leptin [29]	Direct	Activate MMP-13	Promotes OA onset
HTRA1 [30]	Indirect/DDR2	Upregulate MMP-13	Promotes OA onset
LEF1/ELF3/HIF2α/Runx2/CEBPβ [34, 35]	Directly binds to promoter	Upregulate MMP-13	Promotes OA onset
HMW-HA [36, 37]	Indirect	Inhibits production of MMP-13	Inhibits OA onset
miR-9 [41]	Targets MMP-13	Inhibits production of MMP-13	Promotes progression of late-stage OA
miR-146a [42–45]	Targets MMP-13	Inhibits production of MMP-13	Promotes OA onset
miR-127-5p [20]	Targets MMP-13	Inhibits production of MMP-13	Inhibits OA progression
miR-27b [46]	Targets MMP-13	Inhibits production of MMP-13	Inhibits OA progression
miR-320 [47]	Targets MMP-13	Inhibits production of MMP-13	Inhibits OA progression
miR-136 [61]	Targets MMP-13	Inhibits production of MMP-13	Inhibits OA progression
miR-27a [48]	Indirect	Inhibits production of MMP-13	A slight decrease in OA
miR-140 [48]	Indirect/IGFBP-5	Inhibits production of MMP-13	Inhibits OA progression
	Indirect/ER binding miR-140 promoter	Inhibits production of MMP-13	Inhibits OA progression
miR-488 [49]	Indirect/ZIP-8	Inhibits MMP-13 activity	Inhibits OA progression
miR-24 [15]	Indirect	Inhibits production of MMP-13	Inhibits OA progression
miRNA-148a [50]	Indirect	Inhibits production of MMP-13	Inhibits chondrocyte hypertrophy
miR-222 [51]	Indirect/HDAC-4	Inhibits production of MMP-13	Inhibits OA onset
miR-22 [52]	Indirect/BMP-7, PPARα	Activates MMP-13	Promotes OA progression
miR-181b [53]	Indirect	Increases production of MMP-13	Promotes OA progression
miR-33a [54]	Indirect	Increases production of MMP-13	Promotes OA progression
miR-145 [55]	Indirect/Sox9	Increases production of MMP-13	Promotes OA progression
miR-483 [56]	Indirect/BMP-7, TGF-β	Increases production of MMP-13	Promotes OA onset
LncRNA-CIR [59]	Indirect	Increases production of MMP-13	Promotes OA onset and progression
GAS5 [60]	Indirect/miR-21	Increases production of MMP-13	Promotes OA progression
circRNA-CER [61]	Competes with miR-136 as a ‘sponge’	Up-regulation of MMP-13	Promotes OA progression

## Abbreviations

ACLT: Anterior cruciate ligament transaction
ADAMTS: A disintegrin and metalloproteinase with thrombospondin type-1 motifs
ANGPTL4: Angiopoietin-like 4
BMP: Bone morphogenetic protein
circRNA: Circular RNA
CTS: Cyclic tensile stress
ECM: Extracellular matrix
ER: Estrogen receptor
FN-f: fibronectin fragment
HIF: Hypoxia-inducible factor
HMW-HA: High molecular weight hyaluronic acid
HTRA1: High temperature requirement A1
IGFBP: Proanabolic insulin-like growth factor binding protein
K-L grade: Kellgrane and Lawrence grading scale
KO: Knockout
LEF1: Lymphoid enhancer factor-1
lncRNA: Long non-coding RNA
LRP1: Low-density lipoproteinreceptor-related protein 1
MAPK: Mitogen-activated protein kinase
MMP: Matrix metalloproteinase
ncRNA: Non-coding RNA
OA: Osteoarthritis
PPARα: Peroxisome proliferator activated receptor alpha
SIRT1: Silent mating type information regulation 2 homolog 1 (sirtuin-1)
